# Social Identities and Vaccine Exemptions in the United States: The Role of Religiosity, Partisanship, and Vaccine Hesitancy

**DOI:** 10.1007/s10900-026-01597-4

**Published:** 2026-07-23

**Authors:** Matthew R. Boyce, Christine Crudo Blackburn, Syeda Sharmin Duza

**Affiliations:** 1 Department of Health Policy and Management, Texas A&M University School of Public Health, College Station, USA; 2 USA Center for Rural Public Health Preparedness, Texas A&M University School of Public Health, 212 Adriance Lab Road, College Station, TX 77845 USA

**Keywords:** Adolescent health, Child health, Immunization, Public health, Vaccine mandate, Vaccine policy

## Abstract

Nonmedical vaccine exemptions are increasingly common in the United States and threaten population immunity levels in children. While vaccine hesitancy and pediatric vaccination coverage in the United States have been extensively researched, work focused on vaccine exemptions remains relatively limited. The objectives of this study are to examine associations between parental religious, partisan, and vaccine-hesitant social identities and vaccine exemptions. A national, cross-sectional survey was conducted from August 20 through September 9, 2025. Parents and legal guardians of children five years of age or younger were recruited from an online panel and asked if their child had ever missed a vaccination due to a medical or nonmedical vaccine exemption. A total of 1,042 respondents were included in the data analysis, 72 of whom reported that their child did not receive a vaccine due to a nonmedical exemption. The prevalence of nonmedical vaccine exemptions differed significantly according to respondent race, partisanship, religiosity, and vaccine-hesitant identity. Multiple logistic regression modeling produced significant associations between nonmedical exemptions and race, education, religiosity, and vaccine-hesitant identity. These findings reinforce previously identified predictors of vaccine hesitancy in the United States but also extend the evidence base by explicitly examining predictors of vaccine exemptions.

**Supplementary Information:**

The online version contains supplementary material available at https://doi.org/10.1007/s10900-026-01597-4.

## Introduction

Vaccinations remain among the most effective public health interventions and are responsible for averting substantial amounts of preventable morbidity, mortality, and health expenditures. Still, pediatric vaccine rates are declining in the United States (US) [1–3]. In the US, no federal laws currently exist that require pediatric vaccination. However, immunization coverage in pediatric populations has long been promoted at the subnational level by linking school attendance with vaccination. In 1855, Massachusetts became the first state in the United States to make vaccination (i.e., for smallpox) mandatory for entry into public school. Other states gradually followed suit, and by the turn of the twentieth century, most states maintained laws either requiring the vaccination of children attending public schools or allowing local governments to require it [4]. Presently, all 50 states, the District of Columbia, and Puerto Rico require evidence of vaccination against at least some diseases as a condition of entry to public schools [4–6]. 

Still, some children cannot safely receive certain vaccines due to allergies, a history of adverse reactions to vaccines or their components, underlying health conditions (e.g., immunosuppression), or other medical contraindications to vaccinations [6]. Accordingly, children may be exempt from these vaccination requirements due to medical reasons. As of 2019, all 50 states permitted medical vaccine exemptions [CDC, 2022]. Children may also be exempt from vaccination requirements due to nonmedical reasons. These exemptions were initially created to accommodate children of parents belonging to certain religious groups whose teachings objected to vaccines or vaccine ingredients [7]. Presently, policies surrounding these nonmedical vaccine exemptions display considerable heterogeneity between states, but are generally granted on the basis of religion or philosophical beliefs [8]; Hackell et al., 2025]. As of 2019, 46 states permitted religious vaccine exemptions, and 17 states permitted philosophical vaccine exemptions [CDC, 2022]. These nonmedical vaccine exemptions are increasingly common in the United States and threaten population immunity [1–3, 8–12]. For instance, the number of states with exemption rates greater than 5% in kindergartners increased from two in 2020 to 14 in 2023 (i.e., Alaska (9.5%), Arizona (8.5%), Hawaii (5.3%), Idaho (14.3%), Michigan (5.6%), Minnesota (5.4%), Nevada (6.7%), North Dakota (6.4%), Oklahoma (5.6%), Oregon (8.9%), South Dakota (5.7%), Utah (9.3%), Wisconsin (8.0%), and Wyoming (5.6%) [3]. And, critically, nonmedical vaccine exemptions have been linked to outbreaks of vaccine-preventable diseases, such as measles, pertussis, and varicella [13–19].

Notably, nonmedical exemption rates vary considerably within states and across communities, suggesting that exemptions are not randomly distributed within the population [8, 9, 20, 21]. Previous research has found that parental education, trust in government, and trust in science are associated with the odds of nonmedical vaccine exemptions [22–24]. Vaccine exemptions also tend to cluster in specific communities, reflecting underlying social, cultural, and identity-related influences on vaccine-hesitant sentiments [8, 17, 25].

Following this line of reasoning, it is reasonable to predict that certain demographic groups may be more likely than others to pursue nonmedical vaccine exemptions. For instance, while the canonical foundations for declining vaccination are tenuous among major religious traditions [26, 27], previous research has demonstrated that individuals reporting higher levels of religiosity are more skeptical of vaccines [28, 29] and maintain a general distrust of science [30–33]. Others have suggested that purported religious objections to vaccines are, therefore, actually more reflective of concerns about vaccines among a social network of people organized around faith [26]. Others have also suggested that religion may be weaponized when alternate nonmedical exemptions are not available [11, 27, 34]. For example, in one study focused on determining why parents in Massachusetts and Missouriclaim nonmedical exemptions for their children, a minority of parents (i.e., only 8.6% and 22.9%, respectively) indicated religious reasons for forgoing vaccination—this, despite Massachusetts and Missouri authorizing religious reasons as the sole rationale for nonmedical vaccine exemptions [23]. And, unless policymakers are thoughtful in drafting religious exemption legislation, policing whether an exemptor has sincere religious beliefs can be challenging [27].

Beyond religion, however, other population groups may be more likely to seek nonmedical exemptions based on a distrust of science or recommendations from health officials. For instance, the associations between higher levels of trust in science and Democratic political partisanship are well documented [31, 33, 35, 36]. Relatedly, more conservative political views are also a well-documented determinant of anti-vaccination attitudes and beliefs [37–43]. The highly politicized response to the COVID-19 pandemic exacerbated these trends and resulted in disparate attitudes toward vaccines along partisan lines in the United States [43]. And, critically, evidence suggests that sentiments related to COVID-19 vaccines have spilled over toward more routine vaccines [39].

An emerging body of research also suggests that vaccine-hesitant stances have coalesced into a distinct social identity [44–46]. Research has suggested that individuals adopting these social identities tend to hold negative views toward health expertise, evidence-based medicine, and, predictably, vaccines [46, 47]. Accordingly, it is likely that parents who self-identify as “anti-vax” or “vaccine hesitant” would be more likely to pursue nonmedical vaccine exemptions compared to those who do not identify with these groups or labels.

Despite extensive research on vaccine hesitancy and pediatric vaccination coverage in the United States, research focused on vaccine exemptions remains relatively limited [48]. As such, the objectives of this study are to examine the associations between religious, partisan, and vaccine-hesitant social identities and nonmedical vaccine exemptions. Secondary objectives include examining associations between these social identities and medical vaccine exemptions. Acknowledging what is known about these identities and their relationship with trust in science, health expertise, and vaccine attitudes, we hypothesize that nonmedical vaccine exemptions will be more common among parents who identify as more religious, politically conservative, and vaccine-hesitant. We further hypothesize that while these identities will be associated with nonmedical vaccine exemptions, they will not be meaningfully associated with medical vaccine exemptions.

## Methods

### Survey Instrument and Variables

A survey questionnaire was developed to collect information about the prevalence of pediatric vaccine exemptions and parental characteristics. The questionnaire asked respondents if they had a child who had missed a routine vaccination due to a medical exemption, religious exemption, or a non-religious philosophical or moral exemption. Data related to parental characteristics included age, sex (i.e., male or female), race (i.e., American Indian or Alaska Native, Asian, Black or African American, Native Hawaiian or Pacific Islander, White, or other), ethnicity (i.e., Hispanic or non-Hispanic), education (i.e., high school diploma or less, post-high school vocational training, some college (no degree), associate’s degree, bachelor’s degree, or graduate degree), annual household income (i.e., $49,999 or less, $50,000–$99,999, $100,000–$149,999, $150,00–$199,999, or $200,000 or more), partisanship (i.e., strong Democrat, weak Democrat, lean Democrat, Independent, lean Republican, weak Republican, strong Republican), religious denomination (i.e., Buddhist, Catholic, Christian – Protestant, Christian – Orthodox, Christian – Non-denominational, Jewish, Hindu, Mormon, Muslim, other religion, atheist, agnostic, or nothing in particular), religiosity (not religious, very inactive, moderately inactive, neither active nor inactive, moderately active, very active), and self-described vaccine hesitancy (i.e., not hesitant at all, not very hesitant, neither hesitant nor not hesitant, a little hesitant, very hesitant). The survey questionnaire included an attention check to promote data quality. The survey questions are available in an appendix (Supplementary File 1).

### Ethical Review

Study materials were submitted to the Texas A&M University Institutional Review Board for ethical review prior to initiating the recruitment of survey respondents. The research was declared exempt (STUDY2025-0819).

### Recruitment and Data Collection

Eligibility criteria for participation in the survey included being 18 years of age or older, residing in the United States, and being the parent or legal guardian of at least one child 5 years of age or younger. Cint Marketplace was used to recruit survey respondents. Cint sources respondents using a double-opt-in procedure that requires individuals to first opt into the Cint Marketplace platform and then into specific surveys. Cint incentivizes participation in surveys using a points-based system. Survey respondents earn points for completing surveys, which they can later redeem for rewards. The survey questionnaire was administered in English using Qualtrics XM, and data were collected from August 20 to September 9, 2025.

### Data Analysis

1,590 individuals initiated the survey. Of these, 67 individuals (4.2%) were excluded from data analyses for failing to consent to participate, and 228 responses (14.3%) were excluded due to data quality concerns (i.e., failing the attention check completing the survey in less than 4 minutes, etc.). Additionally, 253 (15.9%) respondents who provided incomplete data were also excluded from data analysis. However, dropped responses never exceeded 10% of the total population, removing the need to use imputation methods. This resulted in a population of 1,042 respondents who were included in the data analysis.

Several variables were condensed to facilitate data analysis, and descriptive statistics were used to characterize the study population. Religious vaccine exemptions and non-religious philosophical or moral vaccine exemptions were combined into a single variable (i.e., nonmedical exemptions). Race was collapsed into a nominal, categorical variable by combining American Indian or Alaska Native, Hawaiian or Pacific Islander, and other into a single category (i.e., other). Education was collapsed into a nominal, categorical variable by combining post-high school vocational training and associate’s degree into a single category (i.e., technical certificate or associate’s degree). Partisanship was collapsed into a nominal, categorical variable by combining strong and weak Democrat (i.e., Democrat), lean Democrat, Independent and lean Republican (i.e., Independent), and strong and weak Republican (i.e., Republican). Religiosity was collapsed by combining very inactive and moderately inactive (i.e., inactive), and very active and moderately active (i.e., active).

Religious denomination was omitted from analyses due to issues of collinearity with the religiosity variable. Cross tabulations and chi-squared tests were used to compare the prevalence of medical vaccine exemptions, nonmedical vaccine exemptions, and any vaccine exemption among study subpopulations. Binary logistic regression analysis—including both simple and multiple logistic regression models—was used to examine associations between respondent characteristics and vaccine exemptions. The dependent variables for regression models included medical vaccine exemptions, nonmedical vaccine exemptions, and any vaccine exemption. The independent variables included respondent race, ethnicity, education, income, partisanship, religiosity, and vaccine hesitancy. Models were constructed with robust standard errors, and results are reported as odds ratios with 95% confidence intervals (95% CI) and p-values. The predetermined threshold for statistical significance was set at *p* < .05.

Sensitivity analyses included conducting chi-squared tests and logistic regression analysis with an expanded religiosity variable. StataSE/v19 was used to conduct all data analyses.

## Results

Respondents most often reported being female (*n* = 575, 55.2%), 25–34 years of age (*n* = 398, 38.2%), White (*n* = 661, 63.4%), non-Hispanic (*n* = 850, 81.6%), having attained a high school diploma or less (*n* = 302, 29.0%), an annual household income of $50,000–$99,999 (*n* = 340, 32.6%), a Christian religious denomination (*n* = 493, 47.3%), a Democrat partisan identity (*n* = 470, 45.1%), moderate religious activity (*n* = 309, 29.6%), and not being vaccine hesitant at all (*n* = 293, 28.1%) (Table 1).

**Table 1:** Characteristics of parents and legal guardians of children 5 years of age or younger included in the study population (*n* = 1,042)

Characteristic		No. (%)
Sex		
	Female	575 (55.2)
	Male	467 (44.8)
Age		
	18–24 years	100 (9.6)
	25–34 years	398 (38.2)
	35–44 years	371 (35.6)
	45–54 years	112 (10.7)
	55–64 years	37 (3.5)
	65 years or older	24 (2.3)
Race		
	Asian	104 (10.0)
	Black or African American	170 (16.3)
	White	661 (63.4)
	Other	107 (10.3)
Ethnicity		
	Hispanic	192 (18.4)
	Non-Hispanic	850 (81.6)
Educational attainment		
	High school diploma or less	302 (29.0)
	Some college, no degree	141 (13.5)
	Technical certificate or Associate’s degree	149 (14.3)
	Bachelor’s degree	238 (22.8)
	Graduate degree	212 (20.3)
Annual household income		
	$49,999 or less	335 (32.1)
	$50,000–$99,999	340 (32.6)
	$100,000–$149,999	180 (17.3)
	$150,00–$199,999	109 (10.5)
	$200,000 or more	78 (7.5)
Religion		
	Agnostic, Atheist, or not religious	227 (21.8)
	Buddhist	19 (1.8)
	Catholic	175 (16.8)
	Christian	493 (47.3)
	Jewish	27 (2.6)
	Hindu	7 (0.7)
	Mormon	6 (0.6)
	Muslim	62 (5.9)
	Other	26 (2.5)
Partisanship		
	Democrat	470 (45.1)
	Independent	138 (13.2)
	Republican	434 (41.6)
Religiosity		
	Not religious	227 (21.8)
	Very inactive	62 (5.9)
	Moderately inactive	119 (11.4)
	Neither active nor inactive	123 (11.8)
	Moderately active	309 (29.6)
	Very active	202 (19.4)
Vaccine hesitancy		
	Not hesitant at all	293 (28.1)
	Not very hesitant	205 (19.7)
	Neither hesitant, nor not hesitant	219 (21.0)
	Moderately hesitant	195 (18.7)
	Very hesitant	130 (12.5)
Child has missed a vaccine due to a medical vaccine exemption		
	Yes	59 (5.7)
	No	983 (94.3)
Child has missed a vaccine due to a nonmedical vaccine exemption		
	Yes	72 (6.9)
	No	970 (93.1)

Fifty-nine (5.7%) respondents reported that at least one of their children had missed a vaccine due to a medical exemption, and 72 (6.9%) reported that at least one of their children had missed a vaccine due to a nonmedical exemption. The prevalence of medical vaccine exemptions differed significantly according to respondent education (X^2^ (4, 1,042) = 10.441, *p* = .034), partisanship (X^2^ (2, 1,042) = 7.238, *p* = .027), and religiosity (X^2^ (3, 1,042) = 8.440, *p* = .038) (Table 2). The prevalence of nonmedical vaccine exemptions differed significantly according to respondent race (X^2^ (3, 1,042) = 10.086, *p* = .018), partisanship (X^2^ (2, 1,042) = 25.164, *p* < .001 ), religiosity (X^2^ (3, 1,042) = 14.833, *p* = .002), and vaccine hesitant identity (X^2^ (4, 1,042) = 108.162, *p* < .001). The prevalence of any vaccine exemptions differed significantly according to respondent race (X^2^ (3, 1,042) = 9.659, *p* = .022), education (X^2^ (4, 1,042) = 10.680, *p* = .030), partisanship (X^2^ (2, 1,042) = 23.897, *p* < .001), religiosity (X^2^ (3, 1,042) = 26.341, *p* < .001), and vaccine hesitant identify (X^2^ (4, 1,042) = 100.823, *p* < .001).

**Table 2:** Prevalence of medical, nonmedical, and any type of exemption according to characteristics of parents or legal guardians (*n* = 1,042)

Characteristic		Type of Vaccine Exemption		
		Medical No. (%)	Nonmedical No. (%)	Any No. (%)
Race ^a^				
	Asian (*n* = 104)	4 (3.8)	4 (3.8)	7 (6.7)
	Black or African American (*n* = 170)	6 (3.5)	5 (2.9)	10 (5.9)
	White (*n* = 661)	47 (7.1)	58 (8.8)	79 (11.9)
	Other (*n* = 107)	2 (1.9)	5 (4.7)	6 (5.6)
Ethnicity				
	Hispanic (*n* = 192)	9 (4.7)	15 (7.8)	18 (9.4)
	Non-Hispanic (*n* = 850)	50 (5.9)	57 (6.7)	84 (9.9)
Educational attainment ^b^				
	High school diploma or less (*n* = 302)	23 (7.6)	22 (7.3)	33 (10.9)
	Some college, no degree (*n* = 141)	4 (2.8)	7 (5.0)	10 (7.1)
	Technical certificate or Associate’s degree (*n* = 149)	3 (2.0)	11 (7.4)	13 (8.7)
	Bachelor’s degree (*n* = 238)	10 (4.2)	10 (4.2)	15 (6.3)
	Graduate degree (*n* = 212)	19 (9.0)	22 (10.4)	31 (14.6)
Annual household income				
	$49,999 or less (*n* = 335)	16 (4.8)	16 (4.8)	26 (7.8)
	$50,000–$99,999 (*n* = 340)	16 (4.7)	24 (7.1)	31 (9.1)
	$100,000–$149,999 (*n* = 180)	12 (6.7)	17 (9.4)	21 (11.7)
	$150,00–$199,999 (*n* = 109)	9 (8.2)	9 (8.2)	14 (12.8)
	$200,000 or more (*n* = 78)	6 (7.7)	6 (7.7)	10 (12.8)
Partisanship ^c^				
	Democrat (*n* = 470)	20 (4.2)	19 (4.0)	32 (6.8)
	Independent (*n* = 138)	2 (1.4)	3 (2.2)	5 (3.6)
	Republican (*n* = 434)	36 (8.3)	50 (11.5)	65 (15.0)
Religiosity ^c^				
	Not religious (*n* = 227)	3 (1.3)	4 (1.8)	6 (2.6)
	Inactive (*n* = 181)	8 (4.4)	15 (8.3)	19 (10.5)
	Neither active nor inactive (*n* = 123)	4 (3.2)	6 (4.9)	6 (4.9)
	Active (*n* = 511)	44 (8.6)	47 (9.2)	71 (13.9)
Vaccine hesitancy ^a^				
	Not hesitant at all (*n* = 293)	9 (3.1)	4 (1.4)	13 (4.4)
	Not very hesitant (*n* = 205)	5 (2.4)	7 (3.4)	9 (4.4)
	Neither hesitant, nor not hesitant (*n* = 219)	6 (2.7)	6 (2.7)	9 (4.1)
	Moderately hesitant (*n* = 195)	22 (11.3)	20 (10.2)	30 (15.4)
	Very hesitant (*n* = 130)	17 (13.1)	35 (26.9)	41 (31.5)

^a^ Significant differences for nonmedical vaccine exemptions and any vaccine exemption

^b^ Significant differences for medical vaccine exemptions and any vaccine exemption

^c^ Significant differences for medical vaccine exemptions, nonmedical vaccine exemptions, and any vaccine exemption

Simple logistic regression models suggested that education and religiosity were associated with the odds of a child having a medical vaccine exemption (Table 3). When compared to respondents with a high school diploma or less, the odds were significantly lower for those with a technical certificate or associate’s degree (cOR = 0.25, 95% CI [0.07, 0.93], *p* = .039). And, when compared to those who were not religious, the odds were significantly higher for those who were religiously active (cOR = 4.94, 95% CI [1.43, 7.05], *p* = .012). Neither of these associations remained in the multiple logistic regression model that adjusted for all respondent characteristics (Table 4).

**Table 3:** Crude odds ratios of reporting a medical, nonmedical, or any vaccine exemption (*n* = 1,042)

Characteristic		Crude Odds Ratios [95% CI]		
		Medical Exemption	Nonmedical Exemption	Any Exemption
Race				
	White	Ref.	Ref.	Ref.
	Asian	0.83 [0.25, 2.77]	0.41 [0.15, 1.17]	0.53 [0.24, 1.19]
	Black or African American	0.44 [0.17, 1.14]	*0.31 [0.12, 0.80]	*0.46 [0.23, 0.91]
	Other	0.24 [0.05, 1.08]	0.51 [0.20, 1.30]	0.44 [0.18, 1.03]
Ethnicity				
	Non-Hispanic	Ref.	Ref.	Ref.
	Hispanic	0.80 [0.35, 1.80]	1.18 [0.65, 2.13]	0.94 [0.55, 1.61]
Educational attainment				
	High school diploma or less	Ref.	Ref.	Ref.
	Some college, no degree	0.32 [0.10, 1.01]	0.66 [0.28, 1.59]	0.62 [0.30, 1.30]
	Technical certificate or Associate’s degree	*0.25 [0.07, 0.93]	1.01 [0.48, 2.15]	0.78 [0.40, 1.53]
	Bachelor’s degree	0.74 [0.31, 1.76]	0.56 [0.26, 1.20]	0.55 [0.29, 1.03]
	Graduate degree	1.24 [0.59, 2.62]	1.47 [0.79, 2.74]	1.40 [0.82, 2.36]
Annual household income				
	$49,999 or less	Ref.	Ref.	Ref.
	$50,000–$99,999	1.17 [0.53, 2.58]	1.51 [0.79, 2.90]	1.19 [0.69, 2.05]
	$100,000–$149,999	1.52 [0.64, 3.63]	*2.08 [1.02, 4.22]	1.57 [0.86, 2.88]
	$150,00–$199,999	2.64 [0.97, 7.28]	1.79 [0.77, 4.19]	1.75 [0.88, 3.49]
	$200,000 or more	1.76 [0.58, 5.37]	1.66 [0.63, 4.39]	1.75 [0.80, 3.79]
Partisanship				
	Democrat	Ref.	Ref.	Ref.
	Independent	0.28 [0.06, 1.29]	0.53 [0.15, 1.81]	0.51 [0.20, 1.35]
	Republican	1.59 [0.84, 3.00]	***3.09 [1.79, 5.33]	***2.41 [1.54, 3.76]
Religiosity				
	Not religious	Ref.	Ref.	Ref.
	Inactive	3.41 [0.82, 14.25]	**5.04 [1.64, 15.46]	**4.32 [1.69, 11.06]
	Neither active nor inactive	2.51 [0.50, 12.58]	2.86 [0.79, 10.34]	1.89 [0.59, 5.99]
	Active	* 4.94 [1.43, 7.05]	**5.64 [2.01, 15.88]	***5.94 [2.54, 13.89]
Vaccine hesitancy				
	Not hesitant at all	Ref.	Ref.	Ref.
	Not very hesitant	0.53 [0.15, 1.86]	2.55 [0.74, 8.85]	0.99 [0.41, 2.36]
	Neither hesitant, nor not hesitant	0.48 [0.14, 1.56]	2.03 [0.57, 7.30]	0.92 [0.39, 2.20]
	A little hesitant	1.04 [0.41, 2.66]	***8.26 [2.77, 24.57]	***3.91 [1.99, 7.72]
	Very hesitant	0.71 [0.27, 1.86]	***26.62 [9.22, 76.88]	***9.92 [5.09, 19.35]

* *p* < .05, ** *p* < .01, *** *p* < .001

**Table 4:** Adjusted odds ratios of reporting a medical, nonmedical, or any vaccine exemption (*n* = 1,042)

Characteristic		Adjusted Odds Ratios [95% CI]		
		Medical Exemption	Nonmedical Exemption	Any Exemption
Race				
	White	Ref.	Ref.	Ref.
	Asian	0.75 [0.21, 2.66]	0.72 [0.23, 2.25]	0.89 [0.37, 2.09]
	Black or African American	0.50 [0.17, 1.45]	*0.29 [0.11, 0.77]	*0.40 [0.18, 0.88]
	Other	0.37 [0.04, 3.09]	0.35 [0.10, 1.24]	0.38 [0.12, 1.19]
Ethnicity				
	Non-Hispanic	Ref.	Ref.	Ref.
	Hispanic	0.79 [0.27, 2.35]	1.63 [0.79, 3.38]	1.14 [0.59, 2.20]
Educational attainment				
	High school diploma or less	Ref.	Ref.	Ref.
	Some college, no degree	0.35 [0.09, 1.26]	0.41 [0.15, 1.09]	*0.43 [0.18, 0.99]
	Technical certificate or Associate’s degree	0.28 [0.06, 1.26]	0.61 [0.25, 1.52]	0.51 [0.23, 1.15]
	Bachelor’s degree	0.62 [0.21, 1.81]	*0.34 [0.14, 0.81]	*0.33 [0.15, 0.72]
	Graduate degree	0.85 [0.28, 2.59]	0.51 [0.21, 1.24]	0.55 [0.25, 1.21]
Annual household income				
	$49,999 or less	Ref.	Ref.	Ref.
	$50,000–$99,999	1.44 [0.57, 3.64]	2.10 [0.99, 4.48]	1.60 [0.83, 3.08]
	$100,000–$149,999	1.10 [0.30, 4.04]	2.20 [0.91, 5.30]	1.51 [0.69, 3.30]
	$150,00–$199,999	2.18 [0.60, 7.92]	1.43 [0.46, 4.38]	1.41 [0.55, 3.58]
	$200,000 or more	2.01 [0.53, 7.56]	2.23 [0.71, 7.03]	2.10 [0.81, 5.44]
Partisanship				
	Democrat	Ref.	Ref.	Ref.
	Independent	0.34 [0.07, 1.67]	0.36 [0.09, 1.54]	0.40 [0.13, 1.26]
	Republican	1.22 [0.58, 2.58]	1.41 [0.76, 2.62]	1.18 [0.70, 1.99]
Religiosity				
	Not religious	Ref.	Ref.	Ref.
	Inactive	3.40 [0.66, 17.53]	*3.79 [1.18, 12.18]	* 3.62 [1.36, 9.68]
	Neither active nor inactive	2.43 [0.46, 12.81]	1.58 [0.43, 5.75]	1.22 [0.39, 3.76]
	Active	3.52 [0.98, 12.67]	2.79 [0.90, 8.70]	**3.75 [1.50, 9.38]
Vaccine hesitancy				
	Not hesitant at all	Ref.	Ref.	Ref.
	Not very hesitant	0.77 [0.16, 3.62]	2.38 [0.68, 8.28]	0.94 [0.38, 2.29]
	Neither hesitant, nor not hesitant	0.75 [0.20, 2.84]	2.10 [0.59, 7.41]	1.01 [0.42, 2.39]
	A little hesitant	1.15 [0.38, 3.52]	***7.38 [2.53, 21.56]	***3.62 [1.81, 7.24]
	Very hesitant	0.72 [0.24, 2.16]	***26.66 [9.26, 76.76]	***9.64 [4.79, 19.41]

* *p* < .05, ** *p* < .01, *** *p* < .001

When examining the odds of nonmedical exemptions, simple logistic regression models revealed significant associations with respondent race, income, partisanship, religiosity, and vaccine-hesitant identity (Table 3). Compared to white respondents, the odds of a nonmedical exemption were significantly lower for Black or African American respondents (cOR = 0.31, 95% CI [0.12, 0.80], *p* = .015). Compared to respondents with an annual household income lower than $50,000, the odds were significantly higher for respondents with an annual household income of $100,000–$149,999 (cOR = 2.08, 95% CI [1.02, 4.22], *p* = .043). Compared to Democrats, the odds of a nonmedical exemption were significantly higher for Republicans (cOR = 3.09, 95% CI [1.79, 5.33], *p* < .001). Compared to those who were not religious, the odds were significantly higher for those who were religious but inactive (cOR = 5.04, 95% CI [1.64, 15.46, *p* = .005) and those who were active religiously (cOR = 5.64, 95% CI [2.01, 15.88], *p* = .001). And, compared to those who were not vaccine hesitant at all, the odds were significantly higher for those who were a little vaccine hesitant (cOR = 8.26, 95% CI [2.77, 24.57], *p* < .001) and those who were very vaccine hesitant (cOR = 26.62, 95% CI [9.22, 76.88], *p* < .001).

Significant associations between the odds of a nonmedical exemption and respondent race, religiosity, and vaccine-hesitant identity remained in the multiple logistic regression model (Table 4). In this model, compared to their respective reference groups, the odds were significantly lower for Black or African American respondents (aOR = 0.29, 95% CI [0.11, 0.77], *p* = .013), significantly higher for those who were religious but inactive (aOR = 3.79, 95% CI [1.18, 12.18], *p* = .025), significantly higher for those who were a little vaccine hesitant (aOR = 7.38, 95% CI [0.2.53, 21.56], *p* < .001), and significantly higher for those who were very vaccine hesitant (aOR = 26.66, 95% CI [9.26, 76.76], *p* < .001). Education was also found to be significant in the multiple regression model. Compared to those with a high school diploma or less, the odds of a nonmedical exemption were significantly lower for those with a bachelor’s degree (aOR = 0.34, 95% CI [0.14, 0.81], *p* = .015).

Simple logistic regression models suggested using any type of vaccine exemption as the dependent variable revealed significant associations between vaccine exemptions and race, partisanship, religiosity, and vaccine-hesitant identity (Table 3). Compared to white respondents, the odds of any vaccine exemption were significantly lower for Black or African American respondents (cOR = 0.46, 95% CI [0.23, 0.91], *p* = .026). Compared to Democrats, the odds were significantly higher for Republicans (cOR = 2.41, 95% CI [1.54, 3.76], *p* < .001). Compared to those who were not religious, the odds were significantly higher for those who were religious but inactive (cOR = 4.32, 95% CI [1.69, 11.06, *p* = .002) and those who were active religiously (cOR = 5.94, 95% CI [2.54, 13.89], *p* < .001). And, compared to those who were not vaccine hesitant at all, the odds were significantly higher for those who were a little vaccine hesitant (cOR = 3.91, 95% CI [1.99, 7.72], *p* < .001) and those who were very vaccine hesitant (cOR = 9.92, 95% CI [5.09, 19.35], *p* < .001).

In the multiple logistic regression model, the significance of the associations between the odds of any vaccine exemption and respondent race, religiosity, and vaccine-hesitant identity remained (Table 4). In this model, compared to their respective reference groups, the odds were significantly lower for Black or African American respondents (aOR = 0.40, 95% CI [0.18, 0.88], *p* = .024). The odds were significantly higher for those who were religious but inactive (aOR = 3.62, 95% CI [1.36, 9.68], *p* = .010), those who were religiously active (aOR = 3.75, 95% CI [1.50, 9.38], *p* = .005), those who were a little vaccine hesitant (aOR = 3.62, 95% CI [1.81, 7.24], *p* < .001), and those who were very vaccine hesitant (aOR = 9.64, 95% CI [4.79, 19.41], *p* < .001). Additionally, in this model, compared to respondents with a high school diploma or less, the odds of any vaccine exemption were significantly lower for those with some college but no degree (aOR = 0.43, 95% CI [0.18, 0.99], *p* = .046) and those with a bachelor’s degree (aOR = 0.33, 95% CI [0.15, 0.72], *p* = .005).

Sensitivity analyses using an expanded religiosity variable confirmed these findings (Supplementary File 2). These results revealed that those who were religious but moderately inactive (i.e., as opposed to very inactive) were the primary drivers of associations between vaccine exemptions and respondents who were religious but inactive. These analyses also revealed that the odds of a medical vaccine exemption were significantly higher for those who were moderately religiously active (aOR = 4.01, 95% CI [1.06, 15.14], *p* = .041), when compared to those who were not religious. Additionally, the odds of a nonmedical vaccine exemption were significantly higher for those who were very religiously active (aOR = 3.46, 95% CI [1.07, 11.15], *p* = .038), when compared to those who were not religious.

## Discussion

This study sought to examine the associations between vaccine exemptions and religious, partisan, and vaccine-hesitant social identities in the United States. In line with our *a priori* hypotheses, our findings identify several important predictors of having a nonmedical exemption, but found no significant predictors for medical exemptions. Specifically, our findings suggest that children belonging to White parents, parents with lower levels of education, parents identifying as religious (but not highly active in their religion), and parents with vaccine-hesitant identities were more likely to have nonmedical vaccine exemptions. These findings reinforce previously identified predictors of vaccine hesitancy, but also extend the evidence base in important ways by explicitly examining predictors of vaccine exemptions.

Previous research has found that nonmedical vaccine exemptions tend to be highly clustered geographically [8, 9, 20, 21], and are more likely in areas where a greater proportion of the population identifies as White [49]. Our findings support this result, as children of Black parents were significantly less likely to have missed a vaccine due to a nonmedical vaccine exemption or any type of vaccine exemption, when compared to children of White parents. Still, racial minority populations also tend to report less trust in science and greater levels of vaccine hesitancy [37, 50–55]. The mechanisms underlying this apparent disconnect remain unclear. Acknowledging systemic forces that perpetuate racial disparities in the United States, we suggest that one underlying mechanism may include barriers for Black parents in obtaining nonmedical vaccine exemptions. Still, racial differences were not observed for medical vaccine exemptions. Accordingly, an alternative explanation is the presence of other underlying sociocultural considerations that deter this distrust and uncertainty from translating to the procurement of vaccine exemptions. We suggest that this disconnect represents an area that is deserving of additional research.

Notably, our findings contrast with previous research that found that non-medical exemption rates were associated with parental college education [22]. In our study, higher levels of education, specifically having a bachelor’s degree, were found to be associated with lower odds of children having a vaccine exemption. This discrepancy may be explained by data granularity, as this study relied on individual-level data, and the previous work relied on county-level data. These results may also speak to contemporary trends in the epidemiology of vaccine-preventable diseases, and the growing divide between more and less educated Americans in their relative trust in science [56]. Irrespective of the mechanism of action, our findings are especially important for providing a more nuanced understanding of how parental education is related to the likelihood of children having vaccine exemptions.

One particularly novel finding from this study was the relationship between nonmedical vaccine exemptions and religion. Many studies have found indications that religion is most commonly used as an excuse for non-religious vaccine-hesitant attitudes as opposed to a genuinely religion-based objection [11, 57]. Our findings add an additional layer of understanding to this previous research in that we find that those who identify as religious, but are not the most religiously active, are more likely to have a nonmedical vaccine exemption than the most religiously active. Given that most religions do not prevent or advise against vaccination in their doctrine [26, 27], our findings suggest that those with lower levels of religious activity may be erroneously pointing to religion to support their vaccine-hesitant beliefs, as has been suggested in previous studies [11, 27, 34].

Previous research has explored the influence of vaccine hesitancy in obtaining nonmedical vaccine exemptions, and much policy has been focused on eliminating these opportunities to avoid vaccination. Our findings add to the understanding of the way in which vaccine-hesitant attitudes affect nonmedical vaccine exemptions. Notably, previous research has been mixed regarding the impact of eliminating nonmedical exemptions [58, 59]. Importantly, our findings suggest that vaccine-hesitant attitudes are associated with the likelihood of having a nonmedical exemption, but not a medical exemption, confirming that these two types of exemptions function differently in practice. It is likely that individuals receiving medical exemptions are, in most cases, truly in need of that exemption. In California, where medical exemptions increased when nonmedical exemptions were prohibited, the authors suggested that there was an availability of doctors willing to be flexible about what qualified as a medical exemption [59]. Our findings add support to this interpretation in that they find there is no relationship between vaccine-hesitant attitudes and medical exemptions. We suggest that future research further explore the link between religion and nonmedical vaccine exemptions, given the disconnect between religious teachings on vaccines and the use of religion to obtain nonmedical exemptions. Specifically, further exploration should be done to validate the findings of this study that suggest those who consider themselves religious but are less active in the religion are more likely to have nonmedical vaccine exemptions for their children.

Lastly, our findings contribute to the evidence base related to the relationship between politics, partisanship, and vaccines. As noted earlier, previous work has established that more conservative political views tend to be associated with anti-vaccination attitudes [37–43]. Still, relatively few of these works have also accounted for other social identities that may influence attitudes toward vaccination, such as religion. Callaghan and colleagues found that both conservative ideology and religiosity were significantly associated with not pursuing COVID-19 vaccines due to the perception that they were not safe [37]. While Carroll and colleagues found that Catholic and Evangelical identities were found to predict COVID-19 vaccination rates, these effects became insignificant when controlling for political affiliation [38]. Similarly, in simple regression models in our study, partisanship was found to be an important predictor of nonmedical vaccine exemptions, but the significance of partisanship disappears when adjusting for religiosity and vaccine hesitancy. These findings add further context and understanding to the modern-day relationship between partisanship, religion, and conspiratorial or anti-science beliefs that can often vaccine hesitancy [60].

This study has several limitations that require acknowledgment. First, the cross-sectional nature of our data limits the temporal validity of results and confounds the generalizability of our findings. Furthermore, our study design is unable to account for exemptions from specific routine childhood vaccines. This is notable, as there are relatively higher levels of hesitancy toward certain vaccines in the United States (e.g., those for measles, mumps, and rubella). Future research efforts investigating vaccine exemptions may wish to specify which vaccinations a child is exempt from receiving. Finally, it is important to recognize that our study population may limit generalizability. While online convenience samples are commonly and increasingly used in public health research, they do come with limitations (i.e., requiring internet connection), which could limit the representativeness of the sampling frame. Furthermore, the lack of reliable benchmarks for the study population (i.e., parents of children 5 years of age or younger) limits opportunities to apply weights and adjust for this limitation methodologically. As such, these results should be interpreted as relating to a relatively large and diverse population, but not necessarily representative of the population as a whole.

Despite these limitations, our findings represent a novel contribution to the literature surrounding vaccine hesitancy and add to our understanding of predictors related to nonmedical vaccine exemptions. They also highlight the important differences between medical and nonmedical exemptions—showing that, while there are sociodemographic predictors for nonmedical exemptions, similar predictors were not observed for medical exemptions. This finding suggests that medical exemptions are likely to be based on necessity and are not being used by individuals who are opposed to vaccinations. Identifying and understanding these predictors of vaccine exemptions will be important for creating and targeting public health messaging and education as exemption rates continue to grow and vaccine-preventable diseases continue to spread in the United States.

## Supplementary Information

Below is the link to the electronic supplementary material.

Supplementary Material 1

Supplementary Material 2

## Data Availability

The data that support the findings of this study will be made available from the corresponding author upon reasonable request.
