# Supplementary material for: Social Identities and Vaccine Exemptions in the United States: The Role of Religiosity, Partisanship, and Vaccine Hesitancy
Source: J Community Health. 2026 Jul 23;51(5):865–76. doi: 10.1007/s10900-026-01597-4 (PMC13614974; doi:10.1007/s10900-026-01597-4)
Supplement: Supplementary file 1 [file 10900_2026_1597_MOESM1_ESM.docx]

**Supplement 1. Survey Questions.**

If you wish to participate, please click the "I agree" button and you will be taken to the survey. If you do not wish to participate, please select "I disagree" or select the X in the corner of the window to close your browser.

o I agree

o I disagree

We care about the quality of our survey data. For us to get the most accurate measures of your opinion, it is important that you provide thoughtful answers to the questions in this survey. Do you commit to providing thoughtful answers to the questions in this survey?

o I cannot promise either way

o Yes, I commit to providing thoughtful answers

o No, I will not commit to providing thoughtful answers

**Q.1.** Has your child/children ***missed or not received*** any recommended vaccines?

o Yes

o No

o Unsure

*Display this question if Q.1. = Yes*

**Q.1.1.** If yes, why did they not receive the recommended vaccine? **Select all that apply**.

▢ Was not able to access vaccines/healthcare system (e.g., missed appointment)

▢ Concerns about the safety and/or efficacy of vaccines

▢ Concerns about insurance or ability to pay for vaccines

▢ Exemption – medical (e.g., child is allergic to vaccines)

▢ Exemption – personal beliefs (non-religious philosophical or moral objection to vaccination)

▢ Exemption – religious

▢ I do not think my child needs vaccines

▢ Other

**Q.2.** To let us know that you are still paying attention, please select fruit from the list of foods below.

o Egg

o Blueberry

o Pizza

o Ice cream

o Fish

**Q.3.** Where do you currently reside?

**Q.3.1.** State [Drop Down]

**Q.3.2.** County [Drop Down]

**Q.4.** How do you describe your gender?

o Male/Man

o Female/Woman

o Prefer not to answer

**Q.5.** How old are you (in years)?

o 18–24 years

o 25–34 years

o 35–44 years

o 45–54 years

o 55–64 years

o 65 years and older

o Prefer not to answer

**Q.6.** For this survey, Hispanic, Latino, or Spanish origins are considered an ethnicity and not considered a race. Which race best describes you?

o American Indian or Alaska Native

o Asian

o Black or African American

o Native Hawaiian or Pacific Islander

o White

o Other

o Prefer not to answer

**Q.7.** Are you of Hispanic, Latino, or Spanish origin?

o Yes, I am of Hispanic, Latino, or Spanish origin

o No, not of Hispanic, Latino, or Spanish origin

o Prefer not to answer

**Q.8.** What is the highest level of education you have completed or the highest degree you have received?

o Less than high school diploma

o High school diploma or equivalent

o Post high school vocational training

o Some college, but no degree

o Associate’s degree

o Bachelor's degree

o Graduate or professional degree

o Prefer not to answer

**Q.9.** What is your annual household income?

o $49,999 or less

o $50,000 to $99,999

o $100,000 to $149,999

o $150,000 to $199,999

o $200,000 to $249,999

o $250,000 to $299,999

o $300,000 to $349,999

o $350,000 to $399,999

o $400,000 or more

o Prefer not to answer

**Q.10.** What is your present religion, if any?

o Buddhist

o Catholic

o Christian – Protestant

o Christian – Orthodox (e.g., Greek or Russian Orthodox)

o Christian – Non-denominational

o Jewish

o Hindu

o Mormon

o Muslim

o Other

o Athiest (i.e., I believe there is no God)

o Agnostic (i.e., I do not know if God exists)

o Nothing in particular

**Q.11.** How would you classify your level of involvement with your religion?

o Not religious

o Very inactive

o Moderately inactive

o Neither active nor inactive

o Moderately active

o Very active

**Q.12.** How would you characterize your hesitancy toward vaccines?

o Not hesitant at all

o Not very hesitant

o Neither hesitant nor not hesitant

o A little hesitant

o Very hesitant

o Prefer not to answer

**Q.13.** Generally speaking, do you usually think of yourself as a Republican, a Democrat, or an Independent?

o Strong Democrat

o Weak Democrat

o Lean Democrat

o Independent

o Lean Republican

o Weak Republican

o Strong Republican

o Prefer not to answer

**Q.14.** To let us know that you are still paying attention, please select the baseball team that is from the City of Chicago.

o New York Yankees

o San Francisco Giants

o Texas Rangers

o Chicago Cubs
