# Supplementary material for: Social Identities and Vaccine Exemptions in the United States: The Role of Religiosity, Partisanship, and Vaccine Hesitancy
Source: J Community Health. 2026 Jul 23;51(5):865–76. doi: 10.1007/s10900-026-01597-4 (PMC13614974; doi:10.1007/s10900-026-01597-4)
Supplement: Supplementary file 2 [file 10900_2026_1597_MOESM2_ESM.docx]

**Supplement 2. Supplemental Tables.**

**Table S1.** Prevalence of medical, nonmedical, and any type of exemption according to respondent characteristics.

| Characteristic | | Type of Vaccine Exemption | | |
| --- | --- | --- | --- | --- |
|  |  | Medical  No. (%) | Nonmedical  No. (%) | Any Exemption  No. (%) |
| Race ^a^ | |  |  |  |
|  | Asian (n=104) | 4 (3.8) | 4 (3.8) | 7 (6.7) |
|  | Black or African American (n=170) | 6 (3.5) | 5 (2.9) | 10 (5.9) |
|  | White (n= 661) | 47 (7.1) | 58 (8.8) | 79 (11.9) |
|  | Other (n=107) | 2 (1.9) | 5 (4.7) | 6 (5.6) |
| Ethnicity | |  |  |  |
|  | Hispanic (n=192) | 9 (4.7) | 15 (7.8) | 18 (9.4) |
|  | Non-Hispanic (n=850) | 50 (5.9) | 57 (6.7) | 84 (9.9) |
| Educational attainment ^b^ | |  |  |  |
|  | High school diploma or less (n=302) | 23 (7.6) | 22 (7.3) | 33 (10.9) |
|  | Some college, no degree (n=141) | 4 (2.8) | 7 (5.0) | 10 (7.1) |
|  | Technical certificate or Associate’s degree (n=149) | 3 (2.0) | 11 (7.4) | 13 (8.7) |
|  | Bachelor’s degree (n=238) | 10 (4.2) | 10 (4.2) | 15 (6.3) |
|  | Graduate degree (n=212) | 19 (9.0) | 22 (10.4) | 31 (14.6) |
| Annual household income | |  |  |  |
|  | $49,999 or less (n=335) | 16 (4.8) | 16 (4.8) | 26 (7.8) |
|  | $50,000–$99,999 (n=340) | 16 (4.7) | 24 (7.1) | 31 (9.1) |
|  | $100,000–$149,999 (n=180) | 12 (6.7) | 17 (9.4) | 21 (11.7) |
|  | $150,00–$199,999 (n=109) | 9 (8.2) | 9 (8.2) | 14 (12.8) |
|  | $200,000 or more (n=78) | 6 (7.7) | 6 (7.7) | 10 (12.8) |
| Partisanship ^c^ | |  |  |  |
|  | Democrat (n=470) | 20 (4.2) | 19 (4.0) | 32 (6.8) |
|  | Independent (n=138) | 2 (1.4) | 3 (2.2) | 5 (3.6) |
|  | Republican (n=434) | 36 (8.3) | 50 (11.5) | 65 (15.0) |
| Religiosity ^a^ | |  |  |  |
|  | Not religious (n=227) | 3 (1.3) | 4 (1.8) | 6 (2.6) |
|  | Very inactive (n=62) | 2 (3.2) | 2 (4.2) | 4 (6.4) |
|  | Moderately inactive (n=119) | 6 (5.0) | 13 (10.9) | 15 (12.6) |
|  | Neither active nor inactive (n=123) | 4 (3.2) | 6 (4.9) | 6 (4.9) |
|  | Moderately active (n=309) | 24 (7.8) | 19 (6.1) | 34 (11.0) |
|  | Very active (n=202) | 20 (9.9) | 28 (13.9) | 37 (18.3) |
| Vaccine hesitancy ^a^ | |  |  |  |
|  | Not hesitant at all (n=293) | 9 (3.1) | 4 (1.4) | 13 (4.4) |
|  | Not very hesitant (n=205) | 5 (2.4) | 7 (3.4) | 9 (4.4) |
|  | Neither hesitant, nor not hesitant (n=219) | 6 (2.7) | 6 (2.7) | 9 (4.1) |
|  | Moderately hesitant (n=195) | 22 (11.3) | 20 (10.2) | 30 (15.4) |
|  | Very hesitant (n=130) | 17 (13.1) | 35 (26.9) | 41 (31.5) |

^a^ Significant differences for nonmedical vaccine exemptions and any vaccine exemption.

^b^ Significant differences for medical vaccine exemptions and any vaccine exemption.

^c^ Significant differences for medical vaccine exemptions, nonmedical vaccine exemptions, and any vaccine exemption.

**Table S2.** Crude odds ratios of reporting a medical, nonmedical, or any vaccine exemption.

| Characteristic | | Crude Odds Ratios [95% CI] | | | | |
| --- | --- | --- | --- | --- | --- | --- |
|  |  | Medical  Exemption | | Nonmedical  Exemption | | Any  Exemption |
| Race | |  |  | |  | |
|  | White | Ref. | Ref. | | Ref. | |
|  | Asian | 0.83 [0.25, 2.77] | 0.41 [0.15, 1.17] | | 0.53 [0.24, 1.19] | |
|  | Black or African American | 0.44 [0.17, 1.14] | *0.31 [0.12, 0.80] | | *0.46 [0.23, 0.91] | |
|  | Other | 0.24 [0.05, 1.08] | 0.51 [0.20, 1.30] | | 0.44 [0.18, 1.03] | |
| Ethnicity | |  |  | |  | |
|  | Non-Hispanic | Ref. | Ref. | | Ref. | |
|  | Hispanic | 0.80 [0.35, 1.80] | 1.18 [0.65, 2.13] | | 0.94 [0.55, 1.61] | |
| Educational attainment | |  |  | |  | |
|  | High school diploma or less | Ref. | Ref. | | Ref. | |
|  | Some college, no degree | 0.32 [0.10, 1.01] | 0.66 [0.28, 1.59] | | 0.62 [0.30, 1.30] | |
|  | Technical certificate or Associate’s degree | *0.25 [0.07, 0.93] | 1.01 [0.48, 2.15] | | 0.78 [0.40, 1.53] | |
|  | Bachelor’s degree | 0.74 [0.31, 1.76] | 0.56 [0.26, 1.20] | | 0.55 [0.29, 1.03] | |
|  | Graduate degree | 1.24 [0.59, 2.62] | 1.47 [0.79, 2.74] | | 1.40 [0.82, 2.36] | |
| Annual household income | |  |  | |  | |
|  | $49,999 or less | Ref. | Ref. | | Ref. | |
|  | $50,000–$99,999 | 1.17 [0.53, 2.58] | 1.51 [0.79, 2.90] | | 1.19 [0.69, 2.05] | |
|  | $100,000–$149,999 | 1.52 [0.64, 3.63] | *2.08 [1.02, 4.22] | | 1.57 [0.86, 2.88] | |
|  | $150,00–$199,999 | 2.64 [0.97, 7.28] | 1.79 [0.77, 4.19] | | 1.75 [0.88, 3.49] | |
|  | $200,000 or more | 1.76 [0.58, 5.37] | 1.66 [0.63, 4.39] | | 1.75 [0.80, 3.79] | |
| Partisanship | |  |  | |  | |
|  | Democrat | Ref. | Ref. | | Ref. | |
|  | Independent | 0.28 [0.06, 1.29] | 0.53 [0.15, 1.81] | | 0.51 [0.20, 1.35] | |
|  | Republican | 1.59 [0.84, 3.00] | ***3.09 [1.79, 5.33] | | ***2.41 [1.54, 3.76] | |
| Religiosity | |  |  | |  | |
|  | Not religious | Ref. | Ref. | | Ref. | |
|  | Very inactive | 3.55 [0.48, 26.13] | 1.86 [0.33, 10.40] | | 2.54 [0.69, 9.30] | |
|  | Moderately inactive | 3.37 [0.75, 15.11] | *6.84 [2.18, 21.48] | | **5.31 [2.00, 14.09] | |
|  | Neither active nor inactive | 2.51 [0.50, 12.58] | 2.86 [0.79, 10.34] | | 1.89 [0.59, 5.99] | |
|  | Moderately active | *5.33 [1.48, 19.25] | *3.65 [1.22, 10.89] | | **4.55 [1.88, 11.05] | |
|  | Very active | *4.54 [1.24, 16.60] | ***8.97 [3.09, 26.07] | | ***8.26 [3.40, 20.04] | |
| Vaccine hesitancy | |  |  | |  | |
|  | Not hesitant at all | Ref. | Ref. | | Ref. | |
|  | Not very hesitant | 0.53 [0.15, 1.86] | 2.55 [0.74, 8.85] | | 0.99 [0.41, 2.36] | |
|  | Neither hesitant, nor not hesitant | 0.48 [0.14, 1.56] | 2.03 [0.57, 7.30] | | 0.92 [0.39, 2.20] | |
|  | A little hesitant | 1.04 [0.41, 2.66] | ***8.26 [2.77, 24.57] | | ***3.91 [1.99, 7.72] | |
|  | Very hesitant | 0.71 [0.27, 1.86] | ***26.62 [9.22, 76.88] | | ***9.92 [5.09, 19.35] | |

* *p* < .05, ** *p* < .01, *** *p* < .001

**Table S3.** Adjusted odds ratios of reporting a medical, nonmedical, or any vaccine exemption.

| Characteristic | | Adjusted Odds Ratios [95% CI] | | |
| --- | --- | --- | --- | --- |
|  |  | Medical  Exemption | Nonmedical  Exemption | Any  Exemption |
| Race | |  |  |  |
|  | White | Ref. | Ref. | Ref. |
|  | Asian | 0.71 [0.18, 2.71] | 0.81 [0.26, 2.54] | 0.92 [0.38, 2.22] |
|  | Black or African American | 0.49 [0.17, 1.40] | *0.28 [0.10, 0.73] | *0.39 [0.18, 0.87] |
|  | Other | 0.37 [0.04, 3.38] | 0.31 [0.08, 1.15] | 0.39 [0.11, 1.13] |
| Ethnicity | |  |  |  |
|  | Non-Hispanic | Ref. | Ref. | Ref. |
|  | Hispanic | 0.77 [0.26, 2.29] | 1.73 [0.85, 3.52] | 1.15 [0.60, 2.20] |
| Educational attainment | |  |  |  |
|  | High school diploma or less | Ref. | Ref. | Ref. |
|  | Some college, no degree | 0.34 [0.09, 1.27] | 0.40 [0.15, 1.07] | *0.42 [0.18, 0.97] |
|  | Technical certificate or Associate’s degree | 0.27 [0.06, 1.23] | 0.61 [0.24, 1.54] | 0.52 [0.23, 1.15] |
|  | Bachelor’s degree | 0.63 [0.22, 1.81] | *0.35 [0.15, 0.83] | **0.34 [0.16, 0.72] |
|  | Graduate degree | 0.86 [0.28, 2.66] | 0.52 [0.22, 1.22] | 0.56 [0.26, 1.21] |
| Annual household income | |  |  |  |
|  | $49,999 or less | Ref. | Ref. | Ref. |
|  | $50,000–$99,999 | 1.44 [0.57, 3.64] | *2.19 [1.01, 4.74] | 1.62 [0.83, 3.15] |
|  | $100,000–$149,999 | 1.10 [0.29, 4.07] | 2.05 [0.84, 4.98] | 1.43 [0.66, 3.11] |
|  | $150,00–$199,999 | 2.21 [0.61, 7.99] | 1.48 [0.49, 4.48] | 1.41 [0.56, 3.56] |
|  | $200,000 or more | 2.05 [0.53, 7.87] | 2.13 [0.67, 6.76] | 2.06 [0.79, 5.38] |
| Partisanship | |  |  |  |
|  | Democrat | Ref. | Ref. | Ref. |
|  | Independent | 0.33 [0.07, 1.68] | 0.35 [0.08, 1.46] | 0.39 [0.13, 1.21] |
|  | Republican | 1.25 [0.58, 2.70] | 1.30 [0.70, 2.43] | 1.13 [0.66, 1.91] |
| Religiosity | |  |  |  |
|  | Not religious | Ref. | Ref. | Ref. |
|  | Very inactive | 3.35 [0.40, 28.06] | 1.20 [0.21, 6.98] | 1.85 [0.48, 7.10] |
|  | Moderately inactive | 3.37 [0.55, 20.67] | **5.49 [1.67, 18.00] | **4.81 [1.73, 13.39] |
|  | Neither active, nor inactive | 2.35 [0.45, 12.33] | 1.59 [0.44, 5.80] | 1.23 [0.40, 3.79] |
|  | Moderately active | *4.01 [1.06, 15.14] | 2.31 [0.68, 7.82] | *3.56 [1.34, 9.40] |
|  | Very active | 2.84 [0.74, 10.85] | **3.46 [1.07, 11.15] | **4.06 [1.57, 10.48] |
| Vaccine hesitancy | |  |  |  |
|  | Not hesitant at all | Ref. | Ref. | Ref. |
|  | Not very hesitant | 0.69 [0.14, 3.35] | 2.42 [0.68, 8.60] | 0.92 [0.37, 2.28] |
|  | Neither hesitant, nor not hesitant | 0.73 [0.19, 2.78] | 2.12 [0.59, 7.62] | 1.00 [0.42, 2.40] |
|  | A little hesitant | 1.10 [0.37, 3.28] | ***8.12 [2.76, 23.89] | ***3.75 [1.86, 7.60] |
|  | Very hesitant | 0.77 [0.26, 2.30] | ***26.30 [9.20, 75.15] | ***9.61 [4.75, 19.44] |

* *p* < .05, ** *p* < .01, *** *p* < .001
